# Supplementary material for: Content-rich biological network constructed by mining PubMed abstracts
Source: BMC Bioinformatics. 2004 Oct 8;5:147. doi: 10.1186/1471-2105-5-147 (PMC528731; doi:10.1186/1471-2105-5-147)
Supplement: Additional File 2 — The original results of the above study (non-essential files are deleted to keep the file size under the limit set by BMC bioinformatics). [file 1471-2105-5-147-S2.bz2 › chilibotAdditionalFile2/dip20/761ID8917517E18148/html/DNAB_DNAG.html]

 


 **DNAB** and **DNAG** 
  
Found 91 abstracts in PubMed, retrieved 20.  
 

 What does Google say? 
 PDF only 
| .edu only 

---

**Interactive relationship** (e.g. stimulation, inhibition, etc)

**Neutral relationship**- Mapping protein protein interactions within a stable complex of DNA primase  [ **DNAG** ]  and  **DnaB**  helicase from Bacillus stearothermophilus.  Ref: 10625492 Biochemistry, 2000
- Identification of a region of Escherichia coli  **DnaB**  required for functional interaction with  **DnaG**  at the replication fork.  Ref: 10833513 J Biol Chem, 2000
- We have mapped the surfaces of interaction on both  **DnaG**  and  **DnaB**  and show further that mutations that reduce the physical interation also cause a significant reduction in primer synthesis.  Ref: 8917517 Proc Natl Acad Sci U S A, 1996
- We have shown previously that a protein protein interaction between  **DnaG**  and  **DnaB**  is required to attract the primase to the replication fork.  Ref: 8702920 J Biol Chem, 1996
- Thus, the interaction between  **DnaG**  and  **DnaB**  at the replication fork is the primary regulator of the cycle of Okazaki fragment synthesis.  Ref: 8702921 J Biol Chem, 1996
- Mutations in this region of  **DnaB**  affect the  **DnaB**   **DnaG**  interaction during both general priming directed and phiX174 complementary strand DNA synthesis, as well as at replication forks reconstituted in rolling circle DNA replication reactions.  Ref: 10833513 J Biol Chem, 2000
- Direct physical interaction between  **DnaG**  primase and  **DnaB**  helicase of Escherichia coli is necessary for optimal synthesis of primer RNA.  Ref: 8917517 Proc Natl Acad Sci U S A, 1996
- The primase  **DnaG**  of Escherichia coli requires the participation of the replicative helicase  **DnaB**  for optimal synthesis of primer RNA for lagging strand replication.  Ref: 8917517 Proc Natl Acad Sci U S A, 1996
- As we have demonstrated previously,  **DnaG**  is recruited to the replication fork via a transient protein protein interaction with  **DnaB** .  Ref: 10833513 J Biol Chem, 2000
- ... seven proteins, PriA, PriB, PriC, DnaT,  **DnaB** , DnaC, and  **DnaG** , were required for primosome assembly on phi X174 DNA, only  **DnaB** , DnaC, and  **DnaG**  were required for replication from oriC, suggesting that the other proteins were not involved in chromosomal replication.  Ref: 10506828 Prog Nucleic Acid Res Mol Biol, 1999
- Homogenous assays for Escherichia coli  **DnaB**  stimulated  **DnaG**  primase and  **DnaB**  helicase and their use in screening for chemical inhibitors.  Ref: 12009693 Anal Biochem, 2002

**Non-interactive relationship** (e.g. studied together, co-existance, homology, etc.)

- The initiation proteins of the host,  **DnaB**  and  **DnaG** , and the chaperones DnaJ and DnaK are not required for P4 replication, because polyclonal antibodies against those polypeptides do not inhibit the process.  Ref: 8029013 Nucleic Acids Res, 1994
- RepA protein of pPS10 promotes in vitro plasmid replication in extracts from E. coli, and this replication depends on host proteins DnaA,  **DnaB** ,  **DnaG** , and SSB.  Ref: 7635822 J Bacteriol, 1995
- The  **DnaB**  homolog from Arabidopsis also contains a  **DnaG**  primase domain and the  **DnaB**  homolog from the nematode seems to contain an inactivated version of the primase.  Ref: 10645945 Genome Res, 2000
- Using a combination of biochemical and physical techniques, we show that the helicase primase complex comprises one  **DnaB**  hexamer and either two or three molecules of  **DnaG** .  Ref: 10625492 Biochemistry, 2000
- Kid inhibits in vitro replication of CoEl to a basal level without altering the superhelicity of the template but it does not substantially affect in vitro replication of P4, a DnaA,  **DnaB** , DnaC and  **DnaG**  independent replicon.  Ref: 7723014 J Mol Biol, 1995
- The replication restart proteins, PriA, PriB, PriC,  **DnaB** , DnaC,  **DnaG** , DnaT and Rep, form redundant pathways that recognize repaired replication forks and restart them.  Ref: 11532137 Mol Microbiol, 2001
- For the first time, we demonstrate directly a stable complex between a bacterial  **DnaG**  primase and  **DnaB**  helicase.  Ref: 10625492 Biochemistry, 2000
- The fundamental activities of the replicative primosomes of Escherichia coli are provided by  **DnaB** , the replication fork DNA helicase, and  **DnaG** , the Okazaki fragment primase.  Ref: 10833513 J Biol Chem, 2000
- In Escherichia coli, the primosome assembly proteins, PriA, PriB, PriC, DnaT, DnaC,  **DnaB** , and  **DnaG** , are thought to help to restart DNA replication forks at recombinational intermediates.  Ref: 10835375 Genetics, 2000
- However, recombination was stimulated by mutations in genes encoding replication proteins, including the primosomal proteins  **DnaB** , DnaD and the  **DnaG**  primase, the putative DNA polymerase III subunits PolC, DnaN and DnaX, as well as the DNA polymerase DnaE.  Ref: 11251841 Mol Microbiol, 2001
- In addition, a multicopy  **dnaB**  recombinant, but not a multicopy  **dnaG**  recombinant, prevents the toxicity associated with this protein.  Ref: 7723014 J Mol Biol, 1995
- Gel filtration chromatography was used to isolate both preprimosomal and primosomal complexes formed on single stranded DNA binding protein coated phiX174 DNA by the combination of PriA, PriB, PriC, DnaT,  **DnaB** , DnaC, and  **DnaG** .  Ref: 8663105 J Biol Chem, 1996
- This can be attributed to its ability, unlike wild type DnaC, to catalyze a SSB insensitive general priming reaction with  **DnaB**  and  **DnaG**  on any SSB coated single stranded DNA.  Ref: 10713144 J Biol Chem, 2000
- The behavior of the purified mutant  **DnaB**  proteins in the various replication systems suggests that access to the  **DnaG**  binding pocket on  **DnaB**  may be restricted at the replication fork.  Ref: 10833513 J Biol Chem, 2000
- However, previous studies had not determined whether the activation of the primase or its loading on the template was accomplished by a helicase mediated structural alteration of the single stranded DNA or by a direct physical interaction between the  **DnaB**  and the  **DnaG**  proteins.  Ref: 8917517 Proc Natl Acad Sci U S A, 1996
- The initiation of replication from oriV RSF1010, the replication origin of the broad host range plasmid RSF1010, depends on RepA helicase, RepB primase, and RepC initiator protein, encoded by RSF1010 itself, while this initiation event in E. coli is independent of dnaA,  **dnaB** , dnaC, and  **dnaG**  Scherzinger et al.  Ref: 8690079 FEBS Lett, 1996
- The assay uses an adaptation of the general priming reaction by employing  **DnaG**  primase,  **DnaB**  helicase, and ribonucleotidetriphosphates incorporation of 3 H CTP for in vitro primer synthesis on single stranded oligonucleotide and M13mp18 DNA templates.  Ref: 12009693 Anal Biochem, 2002
